# EGFR-Mediated Carcinoma Cell Metastasis Mediated by Integrin αvβ5 Depends on Activation of c-Src and Cleavage of MUC1

**DOI:** 10.1371/journal.pone.0036753

**Published:** 2012-05-07

**Authors:** Steven K. M. Lau, David J. Shields, Eric A. Murphy, Jay S. Desgrosellier, Sudarshan Anand, Miller Huang, Shumei Kato, Ssang-Taek Lim, Sara M. Weis, Dwayne G. Stupack, David D. Schlaepfer, David A. Cheresh

**Affiliations:** 1 Department of Pathology, Moores University of California San Diego Cancer Center, La Jolla, California, United States of America; 2 Department of Medicine, Moores University of California San Diego Cancer Center, La Jolla, California, United States of America; 3 Department of Reproductive Medicine, Moores University of California San Diego Cancer Center, La Jolla, California, United States of America; Dresden University of Technology, Germany

## Abstract

Receptor tyrosine kinases and integrins play an essential role in tumor cell invasion and metastasis. We previously showed that EGF and other growth factors induce human carcinoma cell invasion and metastasis mediated by integrin αvβ5 that is prevented by Src blockade [1]. MUC1, a transmembrane glycoprotein, is expressed in most epithelial tumors as a heterodimer consisting of an extracellular and a transmembrane subunit. The MUC1 cytoplasmic domain of the transmembrane subunit (MUC1.CD) translocates to the nucleus where it promotes the transcription of a metastatic gene signature associated with epithelial to mesenchymal transition. Here, we demonstrate a requirement for MUC1 in carcinoma cell metastasis dependent on EGFR and Src without affecting primary tumor growth. EGF stimulates Src-dependent MUC1 cleavage and nuclear localization leading to the expression of genes linked to metastasis. Moreover, expression of MUC1.CD results in its nuclear localization and is sufficient for transcription of the metastatic gene signature and tumor cell metastasis. These results demonstrate that EGFR and Src activity contribute to carcinoma cell invasion and metastasis mediated by integrin αvβ5 in part by promoting proteolytic cleavage of MUC1 and highlight the ability of MUC1.CD to promote metastasis in a context-dependent manner. Our findings may have implications for the use and future design of targeted therapies in cancers known to express EGFR, Src, or MUC1.

## Introduction

Epithelial tumor cell metastasis is the culmination of multiple steps including remodeling and invasion of the extracellular matrix [2]. Characterization of the molecular mechanisms coordinating the migration machinery is critical to understanding tumor cell dissemination to secondary sites. In this study, we have identified signaling events that are coordinated by epidermal growth factor (EGF) and a specific integrin to regulate the invasive behavior of human carcinoma cells.

A growing body of literature has revealed that cooperative signaling between receptor tyrosine kinases and integrins regulates cell adhesion, migration, invasion, and survival [3]. In many tumor types, including pancreatic cancer, members of the ErbB family of receptor tyrosine kinases contribute to tumorigenesis and metastasis [4]. We previously reported that integrin αvβ5, in the absence of growth factor stimulation, is unable to form focal adhesions and initiate cell migration/invasion [5]. However, following EGF stimulation, cells expressing integrin αvβ5 gain the ability to invade *in vitro* and metastasize *in vivo* [6], [7] in a Src-dependent manner [1]. In contrast, cell invasion mediated by β1 integrins occurs independent of EGF [6], [7] and Src activity [1]. Understanding how EGFR and Src regulate αvβ5-mediated tumor cell metastasis could lead to novel therapeutic strategies to prevent the metastatic spread of human cancers.

The mucin-1 (MUC1) transmembrane glycoprotein undergoes autocleavage within its SEA (sperm protein-enterokinase-agrin) domain resulting in expression at the cell surface of a stable heterodimer consisting of an extracellular and a transmembrane subunit [8], [9]. MUC1 interacts with ErbB family members including EGFR and is a substrate for Src [10], [11], [12], [13]. MUC1 is overexpressed and aberrantly glycosylated in human neoplasms, particularly adenocarcinomas of the pancreas and breast, and its expression correlates with metastasis [14], [15]. Importantly, the role of MUC1 in metastasis is associated with its intracellular domain, which enters the nucleus and initiates the transcription of a program of genes that modulate tumor metastasis [16], [17], [18], [19].

Here, we show that EGF stimulation promotes MUC1 cleavage, which is both necessary and sufficient to initiate tumor cell metastasis but has no impact on primary tumor growth. Importantly, EGF-induced MUC1 cleavage requires Src kinase activity. These findings identify a pathway of EGF-dependent metastasis that depends on Src-mediated MUC1 cleavage leading to the expression of a metastatic gene signature. These studies may explain in part how inhibitors of EGF or its receptor inhibit the malignant properties of human tumors. Our findings also indicate that Src may be a relevant downstream target for tumors that depend on EGFR signaling and provide rationale for using inhibitors of EGF or its receptors to suppress metastatic disease.

## Results

### MUC1 is required for EGF-induced migration and metastasis

**Figure 1:**
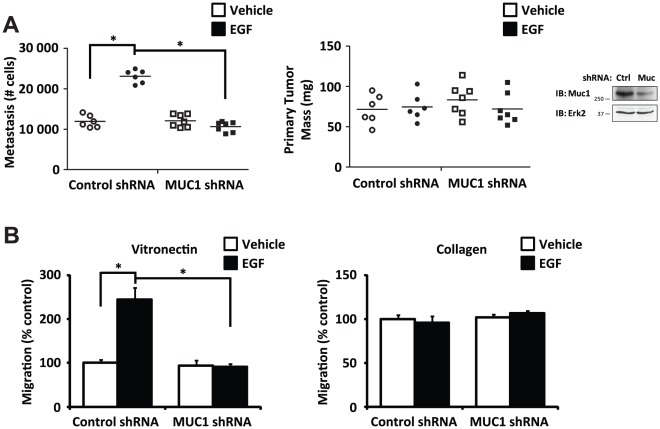
MUC1 is required for EGF-dependent tumor cell metastasis. (**a**) FG human pancreatic carcinoma cells expressing a control shRNA or MUC1 shRNA and stimulated with or without a 15 minute treatment of EGF were inoculated on to the chorioallantoic membrane of 10 day-old embryonated chicken eggs and assessed for spontaneous pulmonary metastasis (left) and primary tumor formation (right) after 10 days. Cells were washed with PBS prior to inoculation to remove EGF. Metastasis quantified by Q-PCR for human *Alu* sequence and chicken *GAPDH* was normalized to a standard curve. Each point represents a separate egg, n≥6 eggs per group. Immunoblot detecting MUC1 expression (far right). *P*<0.0001 comparing metastasis for cells expressing control shRNA with or without EGF treatment; *P* = 0.08 comparing metastasis for cells expressing MUC1 shRNA with or without EGF treatment; *P*<0.0001 comparing metastasis for cells expressing control shRNA or MUC1 shRNA with EGF treatment; *P* = 0.6 comparing primary tumor mass across groups. (**b**) Migration assays on a vitronectin (left) or a collagen (right) substrate comparing FG cells expressing a control shRNA or MUC1 shRNA with or without a 15 minute pre-treatment of EGF. Cells were washed with PBS prior to inoculation on Boyden chambers to remove EGF. *P* = 0.002 comparing migration on vitronectin for cells expressing control shRNA with or without EGF treatment; *P* = 0.8 comparing migration on vitronectin for cells expressing MUC1 shRNA with or without EGF treatment; *P* = 0.002 comparing migration on vitronectin for cells expressing control shRNA or MUC1 shRNA with EGF treatment; *P* = 0.5 comparing migration on collagen across groups. Results are expressed as mean ± s.e.m. of three replicates. Similar findings were observed in 3 independent experiments.

EGFR signaling promotes tumor cell metastasis [20], [21], [22]. Because MUC1 has been shown to interact with EGFR and has been associated with metastasis [10], [23], we assessed its requirement in the well-characterized chick chorioallantoic membrane (CAM) model of EGF-induced metastasis [24], [25]. In this model, FG human pancreatic carcinoma cells are stimulated with a 15 minute treatment of EGF or vehicle *in vitro* and then implanted on the CAM of 10 day-old chick embryos and allowed to spontaneously metastasize to the lungs. After 10 days, primary tumors were weighed and lung metastasis was assessed by Q-PCR. EGF treatment significantly enhanced pulmonary metastasis of FG cells expressing a control shRNA as expected (**Figure 1a**), but shRNA-mediated knockdown of MUC1 expression selectively blocked EGF-induced pulmonary metastasis without preventing primary tumor growth (**Figure 1a**). These data implicate MUC1 as a key regulator of EGF-induced metastasis in this model.

Previous studies have demonstrated that EGF stimulates carcinoma cell metastasis *in vivo*, and this is associated with the induction of integrin αvβ5-mediated cell migration [1], [7]. As such, αvβ5-mediated carcinoma cell migration on a vitronectin substrate *in vitro* may represent a surrogate assay for metastasis *in vivo*. To analyze the role of MUC1 in migration, FG cells subjected to MUC1 knockdown were allowed to migrate on vitronectin or collagen with or without a 15 minute pre-treatment with EGF. EGF-induced migration on vitronectin was abolished by MUC1 knockdown (**Figure 1b**), supporting a role for MUC1 in the EGF-dependent αvβ5-mediated cell migratory response. In contrast, tumor cell migration on collagen was independent of EGF stimulation and MUC1 since knockdown of MUC1 had no effect on this migration response (**Figure 1b**). These findings suggest that MUC1 is required for αvβ5-mediated EGF-induced carcinoma cell migration on vitronectin *in vitro* and spontaneous metastasis *in vivo*.

### EGF treatment promotes nuclear localization of MUC1 and expression of its target genes

**Figure 2:**
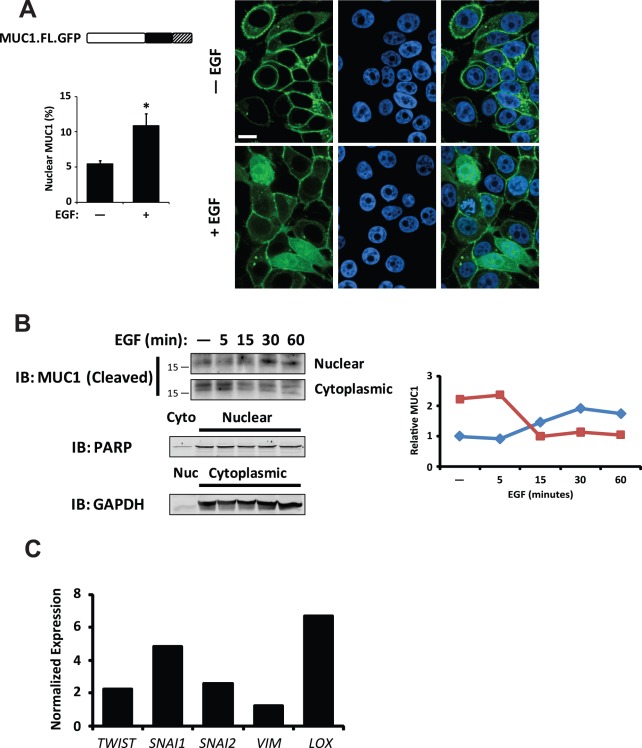
Nuclear localization of MUC1 is EGF-dependent. (**a**) Representative images of immunofluorescence of MUC1.FL fused to GFP (MUC1.FL.GFP; green) with or without a 15 minute pre-treatment with EGF; nuclei are counter-stained with TO-PRO-3 (blue). Schematic (left) illustrates the MUC1.FL.GFP protein product including ectodomain (white), cytoplasmic domain (black) and GFP (hatched). Quantification of nuclear MUC1 (bar graph) is expressed as a percentage of total detectable MUC1. Scale bar represents 10 µm. * *P*<0.0001 compared to unstimulated cells. (**b**) Immunoblot detecting MUC1 cytoplasmic domain showing enrichment of MUC1 cytoplasmic domain with EGF treatment in the nuclear fraction from FG cells. Fraction purity and loading were determined by immunoblotting for PARP (Nuclear, Nuc) and GAPDH (Cytoplasmic, Cyto). Line graph shows quantification of MUC1 in each fraction by densitometry. (**c**) Quantitative RT-PCR of FG cells treated for 15 minutes with EGF compared to untreated control. Peak expression changes over a 24 h period are reported. Values have been normalized to β-actin. Results are expressed as mean ± s.e.m. Similar findings were observed in 3 independent experiments.

The MUC1 cytoplasmic domain has been demonstrated to translocate to the nucleus, where it promotes the transcription of various genes linked to tumor cell invasion and metastasis [17], [19], [26], [27], [28]. Therefore, we considered whether EGF stimulation of FG cells could lead to nuclear translocation of MUC1. To determine whether EGFR signaling induces nuclear translocation of MUC1, we expressed full-length MUC1 (MUC1.FL) fused to GFP and monitored the cells for MUC1 localization by fluorescence microscopy. In the absence of EGF, MUC1.FL localized to the plasma membrane. However, EGF treatment significantly enhanced nuclear localization of MUC1 (**Figure 2a**). To further evaluate the effect of EGF on MUC1 localization in tumor cells, FG cells stimulated with EGF were probed for the MUC1 by immunoblotting. EGF treatment enhanced the amount of MUC1 in the nuclei of these cells within 30 minutes (**Figure 2b**). Importantly, following EGF treatment, we detected elevated transcript levels of several MUC1 target genes linked to tumor cell invasion including *TWIST*, *SNAI1*, and *SNAI2* (**Figure 2c**). Moreover, EGF-induced transcription was abolished by MUC1 knockdown (**Figure S1**), supporting a critical role for MUC1 in the EGF-dependent transcription of genes linked to tumor cell invasion. Together, these results indicate that EGFR signaling promotes translocation of MUC1 to the nucleus, where it regulates transcription of genes linked to invasion and metastasis.

### EGF treatment generates a MUC1 C-terminal fragment that localizes to the nucleus and enhances expression of MUC1 target genes

**Figure 3:**
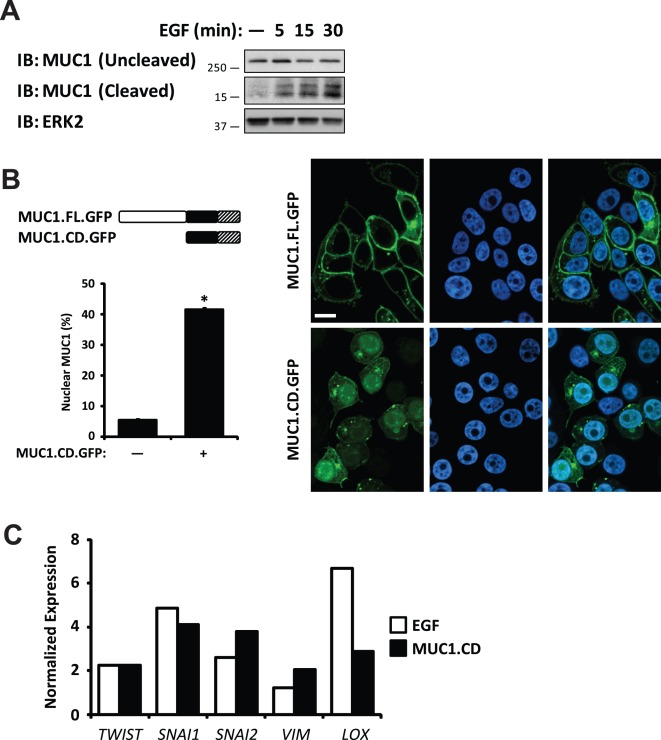
EGF enhances nuclear localization of MUC1 by regulating its cleavage. (**a**) Immunoblot detecting MUC1 cytoplasmic domain showing increased levels of cleavage product with EGF treatment in whole-cell lysates from FG cells. (**b**) Representative images of immunofluorescence of MUC1.FL and MUC1.CD fused to GFP (MUC1.FL.GFP and MUC1.CD.GFP, respectively; green); nuclei are counter-stained with TO-PRO-3 (blue). Schematics (left) illustrate MUC1.FL.GFP and MUC1.CD.GFP protein products including ectodomain (white), cytoplasmic domain (black) and GFP (hatched). Quantification of nuclear MUC1 (bar graph) is expressed as a percentage of total detectable MUC1. Scale bar represents 10 µm. * *P*<0.0001 compared to cells expressing MUC1.FL.GFP. (**c**) Quantitative RT-PCR of FG cells treated for 15 minutes with EGF (white) or expressing MUC1.CD (black) compared to untreated or vector controls, respectively. For cells treated with EGF, peak expression changes over a 24 h period are reported. Values have been normalized to β-actin. Results are expressed as mean ± s.e.m. Similar findings were observed in 3 independent experiments.

Recent studies have demonstrated that the MUC1 cytoplasmic domain plays an important role in tumor cell invasion and anchorage-independent growth [23], [26], [29]. Therefore, we considered whether EGF stimulation of FG cells could lead to cleavage of MUC1. To determine whether EGFR signaling induces cleavage of MUC1, whole cell lysates from FG cells treated with or without a pulse of EGF were probed for the 15 kDa MUC1 cytoplasmic domain by immunoblotting. Within 5 minutes of EGF treatment, we observed increased levels of MUC1 cytoplasmic domain (**Figure 3a**). To assess whether the 72 amino acid MUC1 cytoplasmic domain (MUC1.CD) would localize to the nucleus of carcinoma cells in the absence of EGF, we expressed MUC1.CD fused to GFP and monitored the cells for MUC1 localization. We observed that MUC1.CD localized to the nucleus in the absence of EGF (**Figure 3b**). Furthermore, expression of MUC1.CD enhanced expression of MUC1 target genes linked to metastasis to a similar degree as EGF treatment of FG cells (**Figure 3c**). Together, these results indicate that EGFR signaling promotes cleavage of MUC1 and translocation of the MUC1 cleavage product to the nucleus, where it regulates transcription of a metastatic gene signature.

### The MUC1 cytoplasmic domain is required for EGF-induced migration and sufficient for metastasis

**Figure 4:**
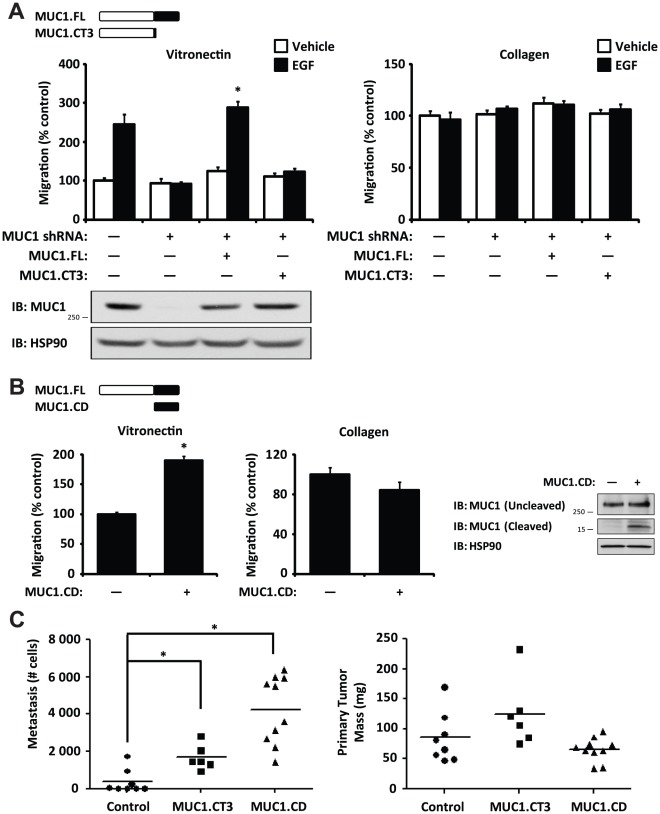
MUC1 cytoplasmic domain is necessary and sufficient for migration and metastasis. (**a**) Migration assays on a vitronectin (left) or a collagen (right) substrate comparing FG cells co-expressing MUC1 shRNA and shRNA-resistant full-length MUC1 (MUC1.FL) or cytoplasmic domain-deleted MUC1 (MUC1.CT3) with or without a 15 minute pre-treatment of EGF. Cells were washed with PBS prior to inoculation on Boyden chambers to remove EGF. Schematic (top) illustration of MUC1.FL and MUC1.CT3 protein products including ectodomain (white) and cytoplasmic domain (black). Immunoblot detecting MUC1 expression with Cell Signaling Technology anti-MUC1 clone VU4H5 (bottom). *P*<0.0001 comparing migration on vitronectin for cells expressing MUC1.FL with or without EGF treatment; *P* = 0.3 comparing migration on vitronectin for cells expressing MUC1.CT3 with or without EGF treatment; *P* = 0.3 comparing migration on collagen across groups. Results are expressed as mean ± s.e.m. of three replicates. Similar findings were observed in 3 independent experiments. (**b**) Migration assays on a vitronectin (left) or a collagen (right) substrate comparing FG cells expressing vector control or MUC1 cytoplasmic domain (MUC1.CD). Schematic (top) illustration of MUC1.FL and MUC1.CD protein products including ectodomain (white) and cytoplasmic domain (black). Immunoblot detecting MUC1 expression (far right). *P* = 0.0003 comparing migration on vitronectin; *P* = 0.2 comparing migration on collagen. Results are expressed as mean ± s.e.m. of three replicates. Similar findings were observed in 3 independent experiments. (**c**) Assessment of spontaneous pulmonary metastasis (left) and primary tumor formation (right) for FG cells expressing vector control, MUC1.CT3, or MUC1.CD in the chick CAM model. Metastasis quantified by Q-PCR for human *Alu* sequence and chicken *GAPDH* was normalized to a standard curve. Each point represents a separate egg, n≥6 eggs per group. * *P*<0.005.

We next asked whether this EGF-dependent MUC1 cleavage product might play a role in EGF-dependent migration. To determine whether the MUC1 cytoplasmic domain was necessary for EGF-dependent migration, FG cells stably expressing MUC1 shRNA were transfected with shRNA-resistant full length MUC1 (MUC1.FL) or cytoplasmic domain-deleted MUC1 (MUC1.CT3) and allowed to migrate with or without a 15 minute pre-treatment with EGF. Whereas knockdown of MUC1 suppressed EGF-mediated cell migration on vitronectin, this response was reversed with MUC1.FL but not MUC1.CT3 (**Figure 4a**). In contrast, expression of either MUC1.FL or MUC1.CT3 did not significantly affect cell migration on collagen, consistent with our findings that MUC1 is not required for EGF-independent cell migration. We next asked whether the MUC1 cytoplasmic domain (MUC1.CD) was sufficient to induce αvβ5-mediated migration on vitronectin of FG cells in the absence of EGF. Interestingly, expression of MUC1.CD in FG cells expressing endogenous MUC1 was sufficient to promote migration on vitronectin without EGF treatment but did not significantly affect migration on collagen (**Figure 4b**).

We then considered whether the MUC1 cytoplasmic domain might be sufficient for tumor cell metastasis. We tested whether expression of MUC1.CD or MUC1.CT3 in FG cells could drive spontaneous pulmonary metastasis in the chick CAM model. Interestingly, expression of MUC1.CT3 significantly enhanced spontaneous pulmonary metastasis compared to control cells (**Figure 4c**). However, MUC1.CD enhanced spontaneous pulmonary metastasis to an even greater degree (**Figure 4c**). Importantly, expression of neither MUC1.CT3 nor MUC1.CD significantly affected primary tumor mass (**Figure 4c**), consistent with our finding that knockdown of MUC1 expression selectively affected metastasis but not primary tumor growth (**Figure 1a**). Together, these results demonstrate that the MUC1 intracellular domain is both necessary for EGF-induced carcinoma cell migration on vitronectin and sufficient to drive cell migration on vitronectin and metastasis in the absence of EGF. In contrast, cell migration on collagen is independent of both EGF and MUC1.

### Src kinase activity is necessary and sufficient for cleavage of MUC1, and MUC1 is required for Src-dependent carcinoma cell migration

**Figure 5:**
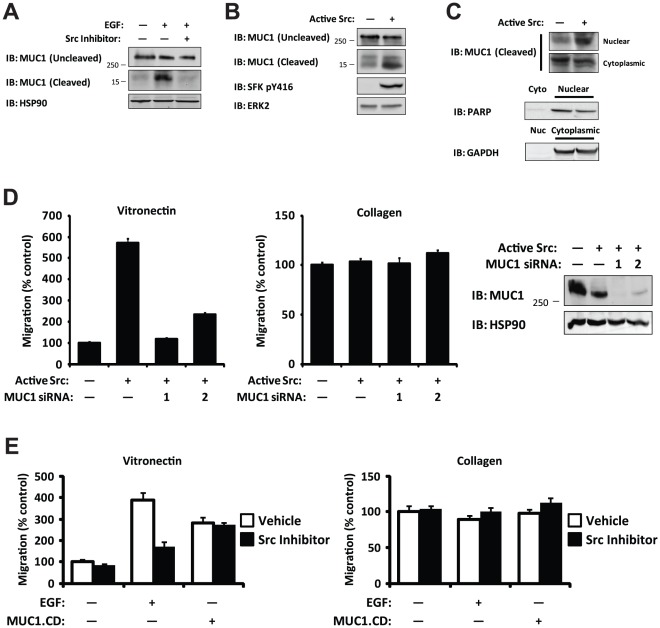
Src activity is necessary and sufficient for MUC1 cleavage, and MUC1 is required for Src-dependent migration. (**a**) Immunoblot detecting MUC1 cytoplasmic domain showing pre-treatment with a Src inhibitor (bosutinib, 500 nM) blocks EGF-dependent MUC1 cleavage in whole-cell lysates from FG cells. (**b**) Immunoblot detecting MUC1 cytoplasmic domain showing increased levels of cleavage product in FG cells expressing constitutively active Src compared to vector control. (**c**) Immunoblot detecting MUC1 cytoplasmic domain showing enrichment of MUC1 cytoplasmic domain in the nuclear fraction from FG cells expressing constitutively active Src compared to vector control. Fraction purity and loading were determined by immunoblotting for PARP (Nuclear, Nuc) and GAPDH (Cytoplasmic, Cyto). (**d**) Migration assays on a vitronectin (left) or a collagen (right) substrate in a Boyden chamber comparing FG cells co-expressing constitutively active Src and either a control siRNA or one of two different MUC1 siRNAs. Immunoblot detecting MUC1 expression (far right). *P*<0.0001 comparing migration on vitronectin for cells expressing vector control or active Src; *P*<0.0001 comparing migration on vitronectin for cells expressing control siRNA or either MUC1 siRNA; *P* = 0.2 comparing migration on collagen across groups. (**e**) Migration assays on a vitronectin (left) or a collagen (right) substrate in a Boyden chamber comparing FG cells expressing either vector control or MUC1.CD with or without a 15 minute pre-treatment of EGF in the presence or absence of a Src inhibitor (bosutinib, 500 nM). *P* = 0.7 comparing migration on vitronectin for cells expressing MUC1.CD with or without Src inhibitor treatment. Similar findings were observed in 3 independent experiments.

EGFR controls various signaling pathways including those of the phosphoinositol-3-kinases, mitogen-activated protein kinases, Janus kinases, and Src family kinases (SFKs) [4]. Importantly, recent studies have demonstrated that activated EGFR recruits and activates SFKs leading to enhanced tumor cell invasion and metastasis [1], [30], [31], [32]. Given that MUC1 is a substrate for Src [11], we considered whether EGF-mediated cleavage of MUC1 was Src-dependent. FG cells were stimulated with a 15 minute treatment of EGF or vehicle in the presence or absence of the Src inhibitor bosutinib and analyzed for the presence of intact or cleaved MUC1. EGF stimulation led to MUC1 cleavage, and this was sensitive to Src inhibition (**Figure 5a**). We next asked whether Src was sufficient to drive cleavage of MUC1 in FG cells. Expression of constitutively active Src in FG cells readily promoted MUC1 cleavage and nuclear localization (**Figures 5b and 5c**). Together, these results demonstrate that Src kinase activity is both required for MUC1 cleavage and sufficient for translocation of the cleavage product to the nucleus.

Since active Src is sufficient for spontaneous migration [1] and our data indicates that Src regulates cleavage of MUC1, we asked whether MUC1 was functioning downstream of Src activity to promote migration. To test whether MUC1 is required for Src-dependent migration, FG cells expressing active Src were transfected with MUC1 siRNA or non-silencing siRNA control. While active Src enhanced αvβ5-mediated migration of cells on vitronectin as expected, knockdown of MUC1 expression with either of two siRNAs selectively blocked Src-dependent migration on vitronectin but had no effect on Src-independent migration on collagen (**Figure 5d**). Interestingly, whereas EGF-induced carcinoma cell migration on vitronectin is sensitive to Src inhibition as expected [1], cell migration driven by the MUC1 cytoplasmic domain does not require Src kinase activity (**Figure 5e**). These data are consistent with the hypothesis that Src activity promotes cell migration by regulating cleavage of MUC1.

## Discussion

Metastasis is responsible for most cancer deaths. Since cross-talk between receptor tyrosine kinases and integrins regulates the metastatic capacity of various human cancers [3], characterization of the molecular mechanisms resulting from this cross-talk is essential to understanding metastasis. Here, we have identified signaling events coordinated by EGFR and integrin αvβ5 that regulate the invasive behavior of human carcinoma cells.

MUC1 expression enhances tumor cell invasion [23], [26], proliferation [33], [34] and survival [35], [36], [37] and is thereby linked to metastasis in several epithelial cancers, including pancreatic and breast carcinomas [14], [15]. As such, MUC1 represents a useful diagnostic and prognostic marker in cancer patients [38], and MUC1 vaccines targeting the ectodomain are currently being studied in ongoing clinical trials [39], [40], [41]. We previously demonstrated coordinated regulation of metastasis by EGFR and integrin αvβ5 [1], [7]. While integrin αvβ5 is unable to initiate cell migration/invasion in the absence of growth factor stimulation [5], stimulation of tumor cells with EGF selectively enhances the ability of cells expressing integrin αvβ5 to invade on vitronectin *in vitro* and metastasize *in vivo* [6], [7]. Moreover, cell migration and metastasis mediated by integrin αvβ5 requires the EGF-dependent activation of Src, which in turn is sufficient for carcinoma cell migration and metastasis [1]. Here, we extend our understanding of this critical signaling pathway by identifying MUC1 as a critical effector of EGF-dependent cell migration and metastasis mediated by integrin αvβ5. Specifically, MUC1 was necessary for EGF-induced migration and metastasis mediated by integrin αvβ5 (**Figure 1**). Furthermore, the MUC1 cytoplasmic domain was both necessary and sufficient to promote migration and metastasis mediated by integrin αvβ5 (**Figure 4**). Significantly, it did not impact migration mediated by β1 integrins (**Figure 4**). To our knowledge, this is the first time that the MUC1 cytoplasmic domain has been demonstrated to play a role in discrete integrin-mediated signaling pathways leading to cell migration and metastasis. As such, antagonists of EGFR or integrin αvβ5 may provide new therapeutic options to target malignant tumors expressing MUC1. Alternatively, it may be possible to target MUC1 with a focused goal of suppressing its cleavage or the function of the cytoplasmic domain.

We have previously utilized FG pancreatic carcinoma cells as a model of the metastatic cascade that is activated downstream of integrin αvβ5, and we have shown that the signaling pathways governing this response in FG cells are representative of those required for invasion and metastasis for a number of additional epithelial cancer types [1], [7], [42]. Thus, the requirement for MUC1 *in vitro* and *in vivo* could represent a general mechanism to drive the metastatic phenotype for carcinoma cells which are dependent upon EGF/EGFR/Src signaling.

Although pharmacologic inhibitors of Src suppress tumor cell metastasis in various animal models [1], [43], [44], [45], [46], the mechanism(s) by which Src contributes to tumor cell metastasis at the molecular level is not yet well-defined and new roles for Src in metastasis are still being described [47], [48]. Our study demonstrates a requirement for MUC1 in Src-dependent migration mediated by integrin αvβ5 (**Figure 5d**). Although MUC1 has been identified as a direct substrate of Src *in vitro* [11], the significance of Src-mediated MUC1 tyrosine phosphorylation is not completely understood but can regulate its association with other proteins including β-catenin and HSP90 [13], [16], [49]. We also observed that EGF treatment induced a Src-dependent MUC1 phosphorylation and that active Src was sufficient for MUC1 phosphorylation (data not shown). However, our findings demonstrate an important and unexpected consequence of interaction between MUC1 and Src: Src activity is critical for MUC1 cleavage (**Figures 5a, 5b**). It is possible that Src-dependent phosphorylation enhances the recognition of MUC1 by a protease. Alternatively, Src may directly or indirectly promote the activity of a protease that constitutively recognizes MUC1. A new series of studies will be required to establish the detailed mechanism by which Src activity enhances MUC1 cleavage.

Accumulating evidence suggests the 72-amino acid cytoplasmic tail of MUC1 regulates a wide array of cellular processes including proliferation, survival, and invasion by modulating signaling pathways at the cell surface, mitochondria, and nucleus. While the precise mechanism of MUC1 cleavage or internalization is not well understood, previous studies have demonstrated trafficking of MUC1 to various subcellular domains in response to receptor tyrosine kinase activation. Importantly, EGF promotes nuclear localization of MUC1 in association with β-catenin [28], [49]. In contrast, the EGF family member HRG promotes nucleolar translocation of MUC1 in association with γ-catenin [50]. Once in the nucleus, MUC1 acts as a co-activator for the expression of genes linked to tumor cell invasion and metastasis including the EMT-promoting genes *TWIST1*, *SNAI1* and *SNAI2* [16], [17], [18], [19]. Moreover, EGF treatment has been demonstrated to enhance transcription of the same EMT-promoting genes [20], [51]. We observed that EGF induced nuclear localization of the MUC1 cytoplasmic domain leading to expression of genes linked to tumor cell invasion and metastasis including *TWIST1*, *SNAI1* and *SNAI2* (**Figure 2c**). This was mimicked by expression of the MUC1 cytoplasmic domain (**Figure 3c**), suggesting that EGF promotes metastasis in part by enhancing MUC1 cleavage leading to a pro-metastatic gene signature.

Expression of EGFR, Src, integrin αvβ5, and MUC1 are associated with the invasive and metastatic potential of various human cancers [3], [15]. Several MUC1 vaccines are currently in phase 3 clinical trials in patients with breast or lung cancer. Furthermore, the MUC1 intracellular domain has become the focus of therapeutic strategies, and MUC1 peptide antagonists have yielded promising results as anti-tumor therapies in mouse models of breast and prostate cancer [52], [53], [54], [55]. Given that the MUC1 cytoplasmic domain is sufficient to enhance metastasis, our studies suggest that therapies targeting the MUC1 ectodomain on tumor cells may provide limited clinical benefit. Importantly, pharmacologic inhibition of Src signaling with bosutinib suppresses MUC1 cleavage, suggesting that targeting the pathways responsible for MUC1 cleavage may represent a promising alternative therapeutic approach. Therefore, therapies suppressing MUC1 cleavage, including antagonists of EGFR and Src, might be beneficial in controlling a wide variety of malignant tumors.

## Materials and Methods

### Antibodies and inhibitors

Antibodies were purchased from Abcam (GFP), BD Biosciences (PARP), Cell Signaling Technology (MUC1 VU4H5, Src pY416), Millipore (GAPDH, Src GD11), Santa Cruz (ERK2, HSP90), Sigma (β-actin), and Thermo Fisher Scientific (MUC1 CT-2). The Src inhibitor bosutinib [56] was used at 500 nM.

### Cell culture

Mycoplasma-negative FG human pancreatic carcinoma cells [57] were grown in DMEM (Life Technologies) supplemented with 10% fetal bovine serum. For some experiments, subconfluent cells were transfected with constitutively active Src (Y527F) in pcDNA3.1 using the Amaxa Nucleofector I (Lonza).

### Short hairpin RNA knockdown

MUC1 and GFP control lentiviral shRNA in pLKO.1 expressing system were from Open Biosystems. Lentiviruses were produced in 293FT cells using FuGene transfection. Cells were selected 48 h after infection with 1 µg/mL puromycin and single-cell clones were isolated, propagated, and screened. For some experiments, subconfluent cells were transfected with MUC1 small interfering RNAs (Qiagen) using Lipofectamine2000 (Life Technologies), and migration assays were performed at 48 h after transfection.

### MUC1 expression

Full length and cytoplasmic domain-deleted MUC1 were generously provided by Michael Hollingsworth [23]. The 3′UTR was removed from these constructs before transfection into cells. The 72 amino acid MUC1 cytoplasmic domain was cloned by PCR and sequenced. MUC1 knockdown cells were transfected with rescue constructs in pcDNA3.1 using Lipofectamine2000 (Life Technologies) and serum starved overnight, and migration assays were performed at 48 h after transfection. For some experiments, the stop codon was removed by site-directed mutagenesis (Agilent Technologies) and the construct was cloned into pGFP (Clontech Laboratories) and then pCDH lentivirus expressing system (System Biosciences). Cells were selected by flow cytometry.

### Protein analysis

Cells were serum starved overnight, pretreated with inhibitors, and stimulated with EGF (50 ng/mL) (Millipore). Immunoblotting and immunofluorescence was performed as previously described [57], [58], [59]. Subcellular fraction was performed as previously described [60]. Images were captured using a TE200E Nikon C1S spectral confocal microscope and analyzed with MetaMorph software (Molecular Devices). Flow cytometry was performed at the UCSD Cancer Center Shared Resource.

### RNA isolation and RT-PCR assays

RNA isolation was performed with Trizol (Life Technologies) according to the manufacturer's instructions. cDNA was generated using the Superscript III First-strand Synthesis Kit (Life Technologies). Reactions containing 200 ng cDNA were prepared in QuantiTect SYBR Green Master Mix (Qiagen) and subjected to quantitative RT-PCR analysis using a Smart Cycler (Cepheid). Values were obtained for the threshold cycle (Ct) for each gene and normalized to β-actin. Values are provided as fold change. For some experiments, cells were treated with EGF (50 ng/mL) for 1, 3, 8, or 24 h before RNA extraction.

### Cell migration and chick embryo metastasis

Migration assays were performed as previously described [57]. Chick embryo metastasis assays were performed as previously described [1]. Briefly, FG cells were stimulated with a 15 minute treatment of EGF or vehicle *in vitro* and then implanted on the chorioallantoic membrane of 10 day-old chick embryos. After 10 days, primary tumors were weighed and spontaneous lung metastasis was assessed by Q-PCR.

### Statistical analysis

Data presented represent mean ± s.e.m. Statistical analyses were performed with Excel (Microsoft) or Prism (GraphPad). Statistical differences for one factor between two groups or more than two groups were determined with an unpaired Student's *t*-test or an analysis of variance (ANOVA) with *post-hoc* testing, respectively. Statistical significance was defined as *P*<0.05.

## Supporting Information

Figure S1 **MUC1 is required for EGF-dependent gene transcription.** Quantitative RT-PCR of FG cells not expressing (white) or expressing (black) MUC1 shRNA and treated for 15 minutes with EGF compared to untreated controls. Peak expression changes over a 24 h period are reported. Values have been normalized to β-actin.
(PDF)
