# Supplementary figures and images for: EGFR-Mediated Carcinoma Cell Metastasis Mediated by Integrin αvβ5 Depends on Activation of c-Src and Cleavage of MUC1
Source: PLoS One. 2012 May 7;7(5):e36753. doi: 10.1371/journal.pone.0036753 (PMC3346745; doi:10.1371/journal.pone.0036753)

**Fig. S1**

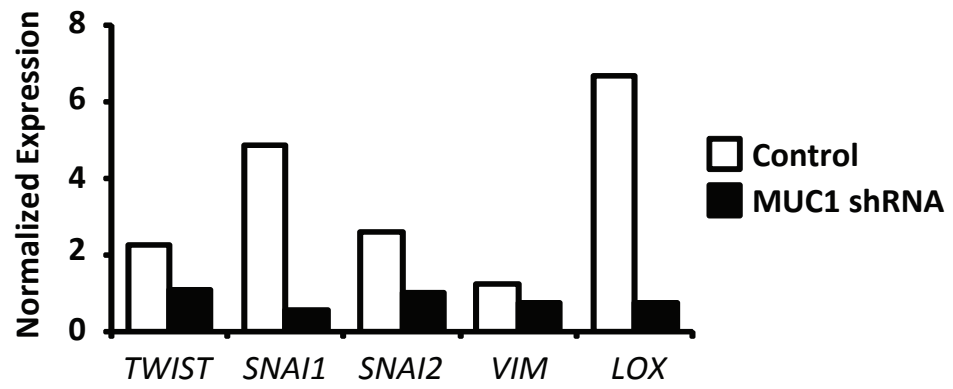

Supplement: Figure S1 — MUC1 is required for EGF-dependent gene transcription. Quantitative RT-PCR of FG cells not expressing (white) or expressing (black) MUC1 shRNA and treated for 15 minutes with EGF compared to untreated controls. Peak expression changes over a 24 h period are reported. Values have been normalized to β-actin. (PDF) [file pone.0036753.s001.pdf]
